# Exploring patient experience of rehabilitation within the surgical pathway for lower limb soft tissue sarcoma in the UK: a single-centre study

**DOI:** 10.1007/s00520-025-09199-x

**Published:** 2025-02-01

**Authors:** Lucy Dean, Siobhan Cowan-Dickie, Dirk C. Strauss, Pauline Humphrey, Fiona Cramp

**Affiliations:** 1 Physiotherapy Department, The Royal Marsden Hospital, Fulham Road, London, SW3 6JJ UK; 2 Sarcoma Unit, Department of Academic Surgery, The Royal Marsden Hospital, Fulham Road, London, SW3 6JJ UK; 3 College of Health, Science and Society, University of the West of England, Blackberry Hill, Bristol, BS16 1DD UK

**Keywords:** Sarcoma, Rehabilitation, Lower limb, Patient experience

## Abstract

**Purpose:**

The primary treatment for localised soft tissue sarcoma (STS) is surgery. Surgery for lower limb sarcoma is associated with poorer functional outcomes than other anatomical sites. Rehabilitation is essential, yet provision is not standardised, and patient experience of current service delivery is unknown. This study therefore aimed to explore patients’ experiences of rehabilitation in the surgical pathway for lower limb STS at a United Kingdom (UK) specialist centre.

**Methods:**

A qualitative, descriptive phenomenological study was undertaken to explore patients’ rehabilitation experiences. Eight patients who had undergone lower limb STS surgery at a specialist centre were purposively sampled. Data were collected through semi-structured interviews and analysed using thematic analysis.

**Results:**

Three main themes were identified: (1) *Accessing the right services at the right time.* Participants described good access to inpatient rehabilitation post-operatively but delays and challenges in accessing local services affected continuity of care. Rehabilitation gaps pre-operatively, and in facilitating return to meaningful activities, were described; (2) “*Communication is key” — providing knowledge and support to navigate uncertainty.* Unclear and unrealistic expectations of recovery were challenging. Communication was key to patients feeling supported and facilitating access to rehabilitation; (3) *The importance of person-centred rehabilitation.* Collaborative, person-centred rehabilitation optimised motivation and engagement.

**Conclusion:**

Participants experienced good access to inpatient rehabilitation post-operatively. In contrast, gaps and delays at other timepoints led to missed opportunities to support preparation for, and recovery from, surgery. A multidisciplinary approach across settings from diagnosis, to deliver person-centred rehabilitation, may improve access, expectation management and continuity of care.

## Introduction

Soft tissue sarcomas (STSs) are a group of cancers arising from the connective tissues and can therefore occur anywhere in the body [1]. Whilst incidence increases with age, STSs are rare, accounting for approximately 1% of adult malignancies [1–3]. The rarity and heterogeneity of STS, which has over 150 histological subtypes [4], mean that it requires specialist management. In the United Kingdom (UK), management is centralised at specialist sarcoma centres (SSCs) [5].

Surgery is the primary treatment for localised STS and may be performed in conjunction with radiotherapy, and less commonly, chemotherapy [1]. Surgery involving connective tissues can result in functional impairments (e.g. muscle weakness), activity limitations (e.g. difficulty walking) and participation restrictions (e.g. work, hobbies) [6–8], with activity levels often remaining reduced in the longer-term [9–11]. The impact of sarcoma and its treatment can adversely affect health-related quality of life (HRQoL) [11–13].

Surgery for lower limb sarcoma is associated with poorer mobility and function and a higher demand for rehabilitation compared with other anatomical sites [14, 15]. However, early post-operative gains in function and activity levels are associated with better long-term function and activity levels [11, 15]. Whilst timely rehabilitation is therefore crucial [6, 7, 16], there are a paucity of studies regarding effective interventions, and rehabilitation provision is not standardised [17]. In current practice at the Royal Marsden Hospital SSC, patients are referred to rehabilitation services pre-operatively when needs are identified. For the majority, rehabilitation commences post-operatively, first on the ward and then with patients’ closest rehabilitation providers.

Previous Australian research reported unmet needs amongst those with sarcoma relating to mobility, daily living, employment, information provision and referral to rehabilitation [18]. Whilst these results cannot be generalised, UK surveys detected low referral rates to rehabilitation, with accompanying narrative suggesting that those who were not referred felt they would benefit [19, 20]. Whilst evidence suggests that patients’ needs are not being met; patients’ experiences of current rehabilitation provision are unknown. Understanding patient experience is critical to developing services and is associated with healthcare quality, better health outcomes and lower health costs [21]. The aim of this study was therefore to explore patient experience of rehabilitation in the surgical pathway for lower limb STS at a SSC. The study objectives were to explore:

- whether rehabilitation had met participants needs and supported a return to meaningful activities;
- whether recovery and rehabilitation had matched any prior expectations;
- experience of continuity of care between rehabilitation services; and
- experience of information provision.

## Methods

### Study design

A qualitative, descriptive phenomenological study was undertaken to explore patients’ rehabilitation experiences. Data were collected through semi-structured interviews with patients who had undergone lower limb STS surgery at a single SSC. Ethical approvals were granted by the Royal Marsden Hospital Service Evaluation Committee (SE1243) and the University of the West of England Research Ethics Committee (HAS.22.12.043). The study was conducted in accordance with the principles outlined in the Declaration of Helsinki. It is reported according to consolidated criteria for reporting qualitative research (COREQ) guidance [22].

### Sample and recruitment

Potential participants were identified from outpatient clinic lists using the eligibility criteria (Table 1). Participants were purposively sampled according to age, gender and distance from the hospital to reflect the clinical population. Potential participants were approached by a member of the sarcoma clinic team (doctors, nurses or physiotherapists) and, if interested, were provided with written information about the study. With patient consent, subsequent email or telephone contact was made by LD, the study lead, to discuss participation. LD is employed by the SSC as a physiotherapist and works within the sarcoma team.

**Table 1:** Eligibility criteria

Inclusion criteria	Exclusion criteria
Participants were eligible if they: (1) were aged 18 years or over; (2) had undergone surgery for lower limb STS involving muscle and/or nerve resection at the SSC between 6 months and 2 years previously; (3) had sufficient mental capacity to understand the purpose of the study and participate; (4) had sufficient English linguistic capability to participate.	Participants were not eligible if they: (1) had undergone amputation; (2) had recurrent or metastatic disease, causing emotional distress whereby recruiting clinicians felt it was insensitive to discuss participation; (3) considered themselves too unwell or burdened to take part.

### Data collection

**Fig. 1:**
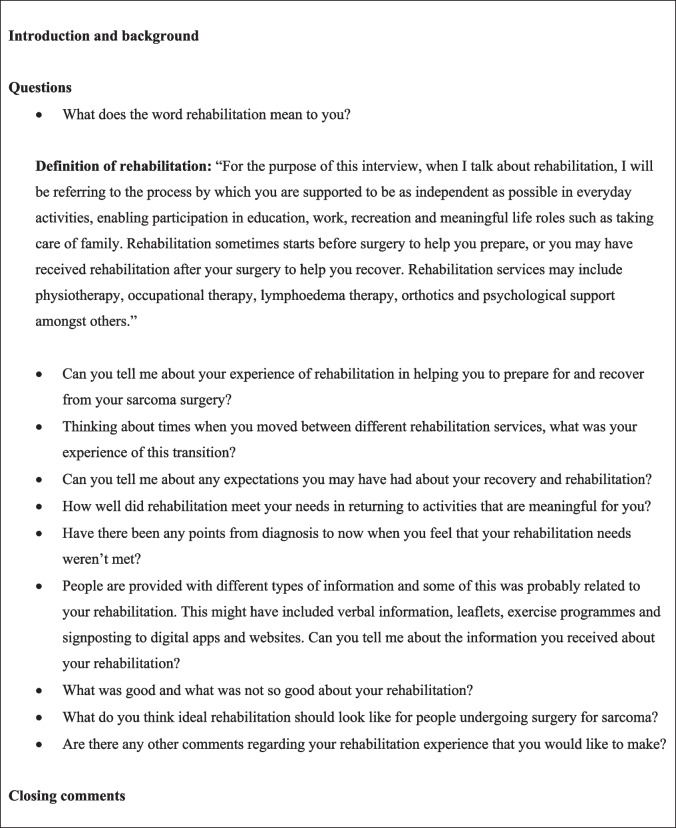
Topic guide summary

All interviews were conducted by LD. The primary platform was online via Microsoft Teams, but telephone and face-to-face interviews were offered as alternatives. Recorded verbal consent was obtained prior to each interview, and participants were informed that they could withdraw their consent up until data anonymisation. Rehabilitation was defined to ensure shared understanding, and a topic guide was used flexibly to allow for discussion of points important to participants (Fig. 1). Topics and questions were guided by the study objectives and based on clinical experience and literature. These were developed collaboratively with three STS patients, a physiotherapist and an occupational therapist at the SSC, and FC and PH (academic supervisors experienced in health/cancer research and qualitative methodologies).

Data collection and analysis took place concurrently, so that emerging topics could be explored. Data collection ended once sufficient information power was achieved. This was defined as the point at which adequate information-rich data had been obtained to produce new knowledge in relation to the study aim, with patterned meaning across the dataset [23]. Interviews were recorded and transcribed verbatim by LD. All quotes were anonymised.

### Analysis

**Fig. 2:**
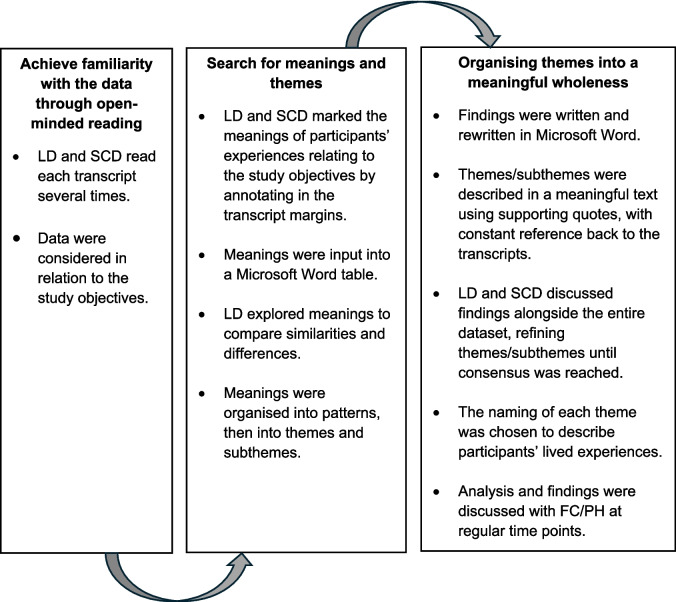
The thematic analysis process using the method described by Sundler et al. [24]

Data were analysed inductively using thematic analysis (TA) for descriptive phenomenology as described by Sundler et al. [24]. The application of this method is shown in Fig. 2. Coding was performed independently by LD and SCD (oncology physiotherapist, external to the sarcoma unit) who read the transcripts several times and annotated in the margins to identify meanings relating to the study objectives. LD explored similarities and differences between meanings, organising meanings into patterns. Patterns were organised into themes and subthemes within a meaningful text to describe the lived experiences of participants. LD and SCD discussed findings alongside the entire dataset, refining themes and subthemes until consensus was reached. TA findings were shared with, and confirmed by, FC/PH.

## Results

**Fig. 3:**
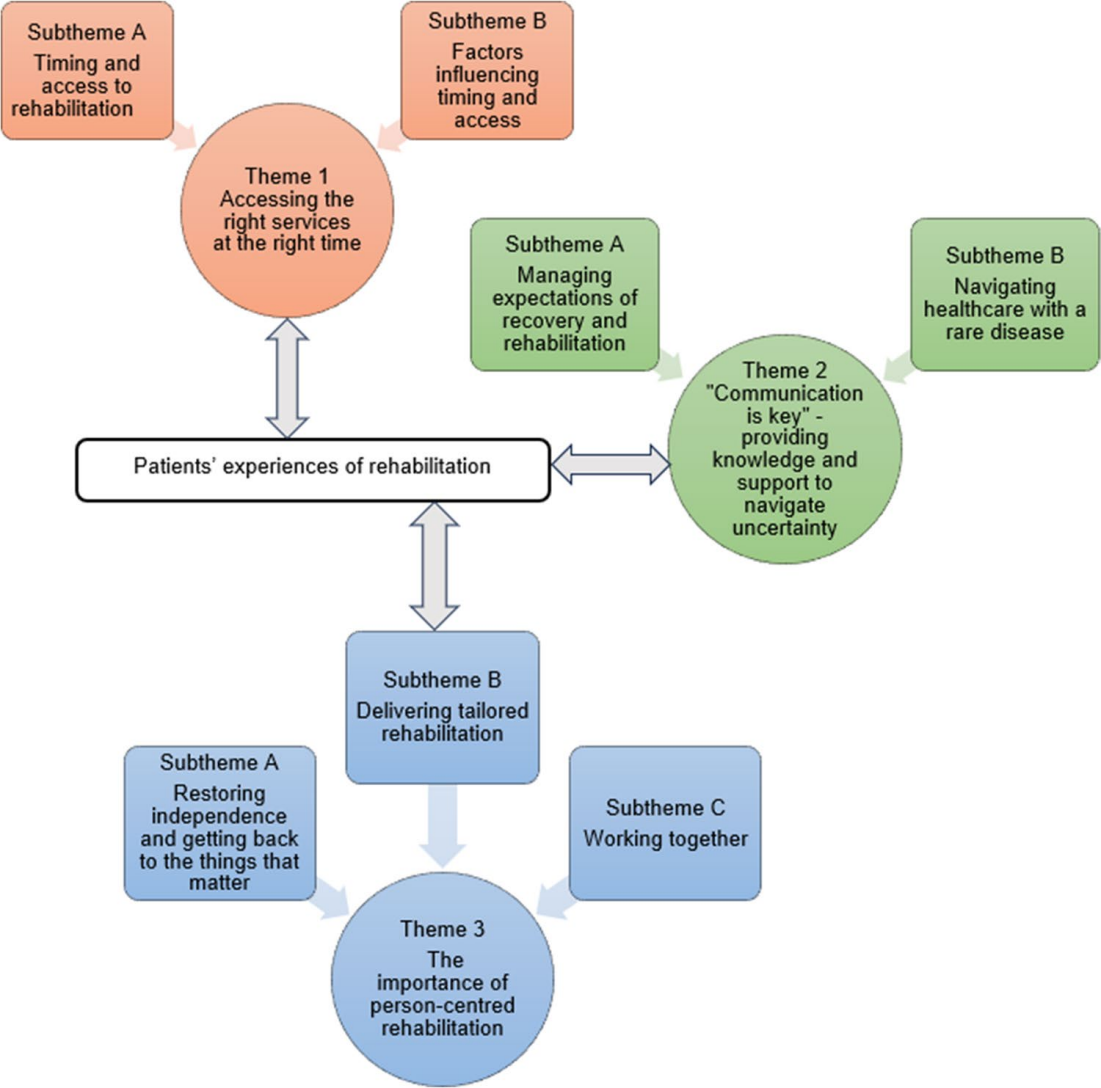
Thematic map

Eight patients were approached during recruitment. All consented to participation. Following a pilot interview, eight semi-structured interviews were conducted between February and April 2023 (Face-to-face *n* = 1; Online via Microsoft Teams *n* = 4; Telephone *n* = 3). Interviews ranged from 45 to 65 minutes. Clinical and sociodemographic participant information is shown in Table 2. Three main themes (and seven subthemes) were identified from TA (Fig. 3):

- Theme 1: Accessing the right services at the right time;
- Theme 2: “Communication is key” — providing knowledge and support to navigate uncertainty; and
- Theme 3: The importance of person-centred rehabilitation.

**Table 2:** Clinical and sociodemographic participant information

Clinical and sociodemographic characteristics	
*N* = 8	
Age at time of surgery, mean (range)	57.8 years (31–79)
Age at time of interview, mean (range)	58.9 years (32–80)
Time since surgery, mean (range)	12.3 months (6–23.5)
Travel distance from the specialist centre in miles, mean (range)	39.5 (1–85)
**Gender, *N***	
Male	4
Female	4
**Ethnicity,* N***	
Asian Indian	1
White British	7
**Employment status,* N***	
Employed full time	1
Employed part time	1
Temporary medical absence from work	1
Unemployed	1
Retired	4
**Tumour site, *N***	
Anterior thigh	1
Antero-lateral thigh	1
Medial thigh	3
Anterior lower leg	1
Posterior lower leg	2
**Sarcoma subtype, *N***	
Well-differentiated/de-differentiated liposarcoma	2
Undifferentiated pleomorphic sarcoma	2
Pleomorphic sarcoma	1
Synovial sarcoma	1
Extraosseous Ewing sarcoma	1
Solitary fibrous tumour	1
**Number of muscles resected, *N***	
< 1	2
> 1 but < 2	1
> 2	4
Whole compartment	1
**Motor nerve involvement, *N***	
None	3
Femoral nerve	1
Obturator nerve	3
Tibial nerve	1
**Plastics reconstruction, *N***	
Yes	1
No	7
**Other treatment received, *N***	
None	2
Pre-operative radiotherapy	4
Post-operative radiotherapy	1
Pre-operative chemotherapy and radiotherapy	1
**Metastatic or recurrent disease, *N***	
Yes	1 (known solitary lung metastasis at surgery)
No	7

### Theme 1: Accessing the right services at the right time

#### Timing and access to rehabilitation

Access to rehabilitation on the ward post-operatively was prompt and co-ordinated.

“One of the things I really thought which helped me most was they were there to help, you know, straight away. It wasn’t like I had to ask for this or ask for that. It was done for me.” (006)

However, participants felt that rehabilitation pre-operatively could have supported physical and psychological preparation.

“I don’t think I was prepared for that operation, but I don’t know how I could have been prepared. I could’ve done some physio and maybe I could have lined up counselling.” (004)

After discharge, some continued their rehabilitation at the SSC, whilst others continued with local rehabilitation providers. The transition to local services was often affected by delays and unclear plans, leaving patients to co-ordinate their rehabilitation at an already challenging time.

“It always seemed to be you’re chasing up your condition… It just felt disconnected... that’s a lot to deal with when you’re dealing with your own stuff.” (008)

This led some to seek support from the SSC or independent services. Physiotherapy review on return to SSC clinics was valued.

“The most important thing to get started was physiotherapy which there was long waiting lists for, so my only contact with physios was when I had clinic appointments. A physio would come to the clinic after I’d seen [surgeon]. It was just a ten/fifteen-minute session but that was really good.” (001)

There were also delays for National Health Service (NHS) psychological support, leading participants to seek alternatives.

“Psychological support took about four months to come through...I had no contact…I did go to [charity], but that was all off my own back.” (001)

Some were discharged from local rehabilitation services prematurely.

“I just felt it wasn’t right to discharge somebody that was three to four weeks out of major surgery…I was still on double crutches. So, my mobility was limited to around the house.” (008)

#### Factors influencing timing and access

Participants were unaware of the rehabilitation available pre-operatively. Post-operatively, whilst timely referrals led to timely rehabilitation where resources were available, late referrals and under-resourced services led to delays.

“The only thing that I was let down on was the community physiotherapy…they were very, very busy and there was only two instead of being five community physiotherapists.” (002)

Medical complications, such as infections, affected timing and access to rehabilitation. Participants also described the challenges of raising and remembering their needs in busy clinics which were often medically-focused.

“As is so often the case, you think, ‘oh crikey, why didn’t I ask that?’” (005)

Participants did not always feel they could discuss their rehabilitation needs with medical professionals.

“I don’t really feel I could ask my consultant, ‘can you arrange physio?’ I felt that she’s more for the cancer side.” (007)

The same participant sought physiotherapy referral via a nurse at their local hospital and was advised that “physio can’t do a lot for you”*,* demonstrating that healthcare professionals’ awareness of the indications for rehabilitation can influence access.

### Theme 2: “Communication is key” — providing knowledge and support to navigate uncertainty

#### Managing expectations of recovery and rehabilitation

Whilst participants felt well-informed about their surgery, unclear and unrealistic expectations about recovery and rehabilitation were common.

“I was told [recovery would be] 4 to 6 weeks…Well that was the understatement of the century.” (001)

Expectations were informed by medical professionals, prior healthcare experiences and information from other sources including the internet. Whilst the challenges of managing expectations were acknowledged, not meeting anticipated recovery timeframes, and the unrealistic expectations of others, negatively affected wellbeing.

“I got all this off the internet. Maybe there should be a sheet to say, some people take longer than others and don’t get too upset if you haven’t achieved it, because it knocks you back.” (004)

This highlights patients’ need for clearer communication and information provision, which was valued by those who received it.

“[Surgeon] probably set me up before surgery to say, ‘this will be a long-term thing, and you may not fully have the mobility you had before surgery.’” (008)

Expectations evolved over time in response to guidance, progress and setbacks. Working with an exercise specialist supported one participant to surpass their expectations.

“I never thought I’d be able to go back to badminton and that, but now there’s a chance I will. I’m like 75% there with this gym [programme]. I didn’t expect to be doing what I’m doing now. So, that’s why I’m so happy.” (p006)

#### Navigating healthcare with a rare disease

Participants described the unfamiliarity of local healthcare services with STS.

“People don’t know about sarcoma. Hospitals don’t, wherever I go. The physio yesterday said ‘Oh, it’s really interesting.’ Of course it is, because it’s not common and no one knows about sarcoma.”(007)

Detailed referral letters to rehabilitation providers and responsive rehabilitation contacts at the SSC, were crucial to patients’ feeling supported through continuity of care.

“It was the fact I knew I could pick up the phone, ring, and I would get a reply that day. If it was late afternoon, it would be first thing the next morning. I followed all the advice I was given.” (002)

### Theme 3: The importance of person-centred rehabilitation

#### Restoring independence and getting back to the things that matter

Restoring independence, reducing reliance on others and returning to meaningful activities were key drivers for engaging in rehabilitation.

“As soon as I could be walking, looking after myself to go to the loo and whatever. That’s all I wanted mainly, is being independent.” (006)

This was optimised by multidisciplinary working.

“...the OT, the physiotherapist, the pain clinic. They work together to help me be more independent.” (003)

The challenges in returning to life roles, driving, work and physical activity alongside the impact of this, were described.

“I thought I’d be back to work by now, I’d like to go back. I think for your head it would be better…I’m up and down the stairs, taking things to classes, nipping up the bank. It’s things that usually take two minutes, and now it’ll take like twenty.” (007)

#### Delivering tailored rehabilitation

Tailored interventions and re-creating home environments and scenarios increased confidence and readiness for discharge post-operatively.

“I was able to do it [stairs] because I prepared myself at the hospital with physios. I’m sure you know that they practice. They have practice steps inside the hospital. So, I kind of got the hang of the basics of walking, one step at a time.” (005)

Personalised goals and programmes, contextualised within prior treatment, increased engagement and were viewed more positively than those which were therapist-led or generic.

“My first goal was driving again. That was a big thing. It was definitely tailored working towards, right, what muscles do I need? What do I need to be able to do to get to that goal?” (001)

Seeing progress provided hope and motivation, with formal assessments and written programmes providing additional direction and accountability.

“I have to keep doing the exercises because he measured how I was doing. And he said, ‘Oh, I’m amazed how well you have done up to now.’ So, it was good, you know? It was worth it. And it gave me more, what do you call it? Enthusiasm to carry on, you know? Motivation.” (006)

The need for tailored rehabilitation was reinforced by participants describing factors that influenced their perceived support needs including self-efficacy, intrinsic motivation and the role of family.

“My wife is my rock. Sometimes she’s also the person that says ‘come on, let’s not sit around and mope around. Let’s try and get going’… I’m also a little bit more self-motivated, to get up, get out, get on with it as much as I possibly could...” (008)

#### Working together

A good patient-therapist relationship contributed to participants feeling supported, enabling them to share their needs and concerns.

“It’s been more than the physio side. It’s being able to come and talk through the stress that’s going on in my head.” (001)

The therapeutic relationship also fuelled motivation.

“It makes you feel like they’re just as invested in your health and your recovery as you are. So, it makes you feel better, which then makes it feel more important to help prove them right.”(003)

Active participation in rehabilitation planning, including decision-making regarding discharge, ensured that participants felt supported whilst building confidence to self-manage.

“I haven’t been pressured into thinking I’ve only been given six sessions or ten sessions, it was just open-ended until I feel, or we both felt, that it’s got to a point, right, I probably don’t need to come anymore. So, that’s taken the pressure off.” (001)

## Discussion

This qualitative study aimed to explore patients’ experiences of rehabilitation within the surgical pathway for lower limb STS at a UK SSC. Findings demonstrate good access to inpatient rehabilitation post-operatively; however, gaps and delays at other timepoints led to missed opportunities to support preparation for, and recovery from, surgery. Less positive experiences are related to poor continuity of care when transferring to local rehabilitation services and managing expectations of recovery. Good communication within a collaborative, person-centred approach to rehabilitation optimised engagement and rehabilitation experience.

Prompt rehabilitation on the ward was valued by participants. It is also important to minimising length of stay [25]. Whilst the benefits of pre-operative rehabilitation, or prehabilitation, are well-evidenced in common cancers [26, 27], research in STS is lacking. Consequently, rehabilitation resource at the SSC is largely focused on the post-operative pathway. Importantly however, participants felt that prehabilitation could have optimised their physical and psychological readiness for surgery. Alongside findings relating to the often unclear and unrealistic expectations of recovery, prehabilitation may also provide an opportunity to improve information provision and inform expectations. Information gaps early in the pathway are consistent with previous findings [18], with one study reporting that those with unclear expectations and those expecting a difficult recovery had worse functional outcomes than those anticipating an easy recovery following lower limb sarcoma surgery [28]. Despite the challenges of managing expectations, acknowledged by participants, specialist allied health professionals (AHPs) are well-placed to address this and should be involved in early conversations regarding recovery.

Participants described delays and challenges in accessing local rehabilitation services post-operatively. This is concerning, particularly as sarcoma is associated with high levels of physical disability compared with other cancers [29], and reduced physical function negatively impacts psychosocial wellbeing [13]. Compounding these difficulties were delays in accessing psychological support; a likely contributor to previous national survey findings whereby only 18% of respondents felt they had ‘definitely received enough emotional support’ [19]. Participants described various factors which influenced their ability to access rehabilitation, including under-resourced community services, a lack of sarcoma awareness external to the SSC, and the development of post-operative complications. The latter finding likely contributes to the association between complications and poorer functional outcomes [30]. Whilst some patients sought interim support from the SSC or independent rehabilitation providers, these solutions are not accessible for all. Rather, the inequity in service provision may perpetuate disability and health inequalities, posing a threat to addressing priorities outlined in the NHS Long Term Plan [31].

With research suggesting that early post-operative functional gains are indicative of better long-term function [11], rehabilitation delays are likely to be detrimental to overall recovery. Early rehabilitation referrals and rehabilitation plans to bridge the gap between services are therefore crucial. Findings from this study, including the difficulties faced by participants in raising their needs, highlight the need for a more proactive approach to healthcare. This is consistent with previous recommendations to embed regular holistic needs assessments (HNAs) from diagnosis [32–34]. Integrated multidisciplinary team (MDT) working including rehabilitation representation at MDT meetings is also recommended and could help identify those at high-risk of complications and poor functional outcomes. The results highlight the important role of AHPs in facilitating access to rehabilitation. Good communication between services and being able to contact the SSC rehabilitation team were essential to participants feeling supported. Detailed referral letters were also valued, with the inclusion of information such as expectations of recovery particularly important given the rarity of sarcoma.

Findings show that person-centred rehabilitation was important throughout the pathway, from optimising readiness for hospital discharge to the consideration of patients’ drivers, barriers and enablers to rehabilitation. Personalised goals and programmes, contextualised within prior treatment, also optimised engagement. These findings support previous recommendations to utilise the International Classification of Functioning, Disability and Health (ICF) framework to address the multidimensional needs of those with sarcoma [6, 7, 35]. To deliver personalised care, this study also highlights the importance of involving patients in decision-making; echoed within the literature, NHS priorities and NICE guidelines [36–38]. The therapeutic relationship enabled participants to share their needs and concerns. In the context of scarce access to psychological support, this is important given that unmet needs are associated with reduced HRQoL [12].

Restricted participation in life roles and situations has the greatest impact upon HRQoL [39]. It is therefore unsurprising that returning to meaningful activities was a key motivator amongst participants. Consistent with previous findings, however, were the challenges described in returning to activities including work, driving, life roles and physical activity [7, 9, 10, 40]. Alongside physical disability, this may be influenced by the finding that discharge from community rehabilitation services can be premature. Given the importance of activities such as work and physical activity [40, 41], tailored support in later recovery is essential and was viewed positively by those that had access. Being supported to return to badminton through a supervised exercise programme led one participant to surpass their expectations of recovery. Whilst participants predominantly accessed physiotherapy, the challenges faced in returning to work signify the importance of better access to services including occupational therapy and vocational rehabilitation. In addition to addressing physical impairments, it is imperative to address the psychological challenges associated with returning to work [40]. With previous studies demonstrating the impact of sarcoma on physical and psychosocial functioning, this study provides a single centre’s insight into the impact of rehabilitation provision on those domains.

Limitations of this study include its small, single-centre sample. However, findings are likely to be transferable to similar settings and contexts. Most participants underwent extensive surgery, resulting in automatic physiotherapy review. This study is not, therefore, representative of those undergoing smaller day-case procedures following which, physiotherapy assessment is not routine. The predominant service accessed after hospital discharge was physiotherapy and a bigger sample could have increased diversity in the services required. Some participants may have received rehabilitation whilst COVID-19 restrictions were in place which may have influenced access. The time since surgery was considered in eligibility criteria selection; however, recall bias cannot be eliminated [42]. Whilst the sample was representative in terms of gender and distance from the SSC, a short recruitment timeframe resulted in poor ethnic diversity and under-representation of those aged between 18 and 31 years. The lack of access to translation services excluded non-English speakers who may be disadvantaged in terms of access to healthcare services [43]. Thus, health inequalities may not be adequately reflected. The small sarcoma rehabilitation team means that several participants had clinical contact with the study lead. However, transparency and reflexivity were employed throughout to minimise bias [24].

Findings from this study should be used to inform future multi-centre research exploring patients’ experiences of rehabilitation in STS. Intervention studies to identify effective models of prehabilitation and rehabilitation in this population are warranted.

## Conclusion

Despite timely inpatient rehabilitation post-operatively, gaps and delays at other timepoints led to missed opportunities to support preparation for, and recovery from, surgery. The lack of research into prehabilitation in STS means that it is not currently embedded in practice. However, with participants describing a need for earlier rehabilitation alongside the often unclear and unrealistic expectations of recovery, research in this area should be prioritised. A more proactive approach to rehabilitation could be achieved by embedding regular HNAs alongside truly integrated MDT working, with rehabilitation representation at MDT meetings. To improve continuity of care in rehabilitation, prompt and detailed referrals should be provided for professionals, alongside rehabilitation plans and key contact details for patients. Finally, a person-centred approach to rehabilitation optimised patient engagement and experience, signifying its fundamental importance to patient outcomes.

## Data Availability

Anonymised data are held securely and are not publicly available in order to protect participant privacy. Anonymised clinical and sociodemographic participant information, and excerpts of anonymised transcripts relevant to the findings presented in this manuscript, can be made available by the corresponding author upon reasonable request.
